# A dualist theory of experience

**DOI:** 10.1007/s11098-025-02290-3

**Published:** 2025-02-18

**Authors:** Bradford Saad

**Affiliations:** Global Priorities Institute, University of Oxford, Oxford, UK

**Keywords:** Dualism, Consciousness, Interactionism, Non-reductive functionalism, Psychophysical laws, Mental causation, Causal closure, The causal exclusion problem, Overdetermination, Preemption, Organizational invariance

## Abstract

Dualism holds that experiences somehow arise from physical states, despite being neither identical with nor grounded in such states. This paper motivates a stringent set of constraints on constructing a dualist theory of experience. To meet the constraints, a dualist theory must: (1) construe experiences as causes of physical effects, (2) ensure that experiences do not cause observable violations of the causal closure of the physical domain, (3) avoid overdetermination, (4) specify a set of psychophysical laws that yield experiences as a function of physical states, and (5) ensure that functional duplication preserves phenomenology. After motivating these constraints and explaining why existing dualist theories satisfy only some of them, I construct a dualist theory that satisfies all of them. On the resulting theory—which I call *delegatory dualism*—experiences uphold causal responsibilities “delegated” to them by physical states.

## Introduction

*Dualism* holds that experiences somehow arise from physical states, despite being neither identical with nor grounded in such states.^1^ Discussions of dualism tend to focus on arguments for or against it. This paper departs from that tendency with an exercise in theory construction. The exercise is disciplined by a stringent set of constraints. By the end, I will specify a version of dualism that satisfies them. I call the resulting theory *delegatory dualism*. As we will see, delegatory dualism incorporates elements of existing theories that satisfy only some of the constraints.

Here is a sketch of delegatory dualism. Experiences are non-physical states and instances of experiential properties. Experiences inherit their causal profiles^2^ from the experiential properties of which they are instances. Experiential properties have their causal profiles independently of whether they are instantiated. The distribution of experience is settled by the operation of a fundamental psychophysical law. The law dictates that something has a given experiential property whenever that thing has a physical property whose causal profile “matches” that of the experiential property. In addition, another fundamental psychophysical law dictates that, whenever a physical state is accompanied by an experience with a matching causal profile, the physical state “delegates” its causal responsibilities to the experience. On the resulting picture, there is no mystery as to why physical states give rise to experiences with matching causal powers: per the psychophysical law that settles the distribution of experience, physical states give rise to such experiences precisely because this results in such a causal match. Yet overdetermination does not ensue, as physical states uphold their causal responsibilities via delegation rather than direct causation whenever an experience is around that’s qualified for the job.^3^

It’s because delegatory dualism explains the distribution of experience in terms of matching causal profiles between properties that it requires experiential properties to have their causal profiles independently of whether they are instantiated. A corollary of this commitment is that when systems differ in which experiential properties they have, that’s because they have different physical states whose causal profiles correspondingly differ with respect to which experiential properties they match. However, aside from these general commitments, delegatory dualism leaves open which causal profiles are had by which experiential properties. As a result, the theory is compatible with a wide range of views about which causal profiles attach to which experiential properties. This range includes views on which experiential properties’ causal profiles largely cohere with our pre-theoretical beliefs about them. For instance, delegatory dualism can be naturally developed and illustrated with the hypothesis that, at least under suitable circumstances: sensory experiences enable subjects to identify and distinguish objects; valenced experiences (such as pains, pleasures, and emotional experiences) are disposed to cause certain behaviors that are appropriate to the sign and intensity of their valenced component; and various types of experience are reportable and poised to prompt discussion about whether closely related types of experience can be explained in physical terms.^4^

To illustrate delegatory dualism with a toy example, suppose that pain has the (presumably greatly oversimplified) causal profile of being disposed to cause wincing. Further, suppose that a pain causes you to wince. On delegatory dualism, that happens because:

(i) you underwent a physical state that is disposed to cause wincing.
(ii) pain is an experiential property whose causal profile is that of being disposed to cause wincing.
(iii) given this match in causal profiles, a fundamental psychophysical law dictates that a pain accompanies that physical state, and
(iv) given the match in causal profiles, another fundamental psychophysical law dictates that the pain rather than the physical state causes you to wince.

At least on the assumption that experiences have causal profiles like those we pre-theoretically attribute to them, pain and other experiential properties will have causal profiles that are much more complex than that of being disposed to cause wincing. Nonetheless, by substituting experiential properties and their causal profiles (however complex) into the just sketched explanation, delegatory dualism will—in the same manner—explain why those properties are instantiated and why the resulting experiences, rather than their physical bases, cause certain physical effects.

I do not expect delegatory dualism’s appeal to be immediately apparent to anyone from this sketch. Its appeal lies in its distinctive capacity to satisfy the constraints alluded to above. Establishing that capacity is the paper’s main argumentative burden. The result will be a theory that is compatible with what we know and which enjoys both the motivations for those constraints and those provided by arguments for dualism.

Here’s the plan. Section 2 rehearses the much discussed causal exclusion problem for dualism.^5^ That problem suggests three constraints that are jointly inconsistent with dualism: the causal efficacy of experiences, the non-overdetermination of their effects, and the causal closure of the physical domain. Section 3 secures a nearby set of constraints that is consistent with dualism by replacing the causal closure thesis with a weaker claim that captures its empirical content. Section 4 identifies two versions of dualism that live in the logical space defined by those constraints. Section 5 adopts a systematicity constraint that requires dualist theories to specify “upward” psychophysical laws, laws that determine which experiences arise from which physical states. Section 6 addresses a challenge—that of at least providing a toy “downward” psychophysical law, roughly a law that determines what causal contributions experiences inject into the physical domain—that would confront any version of dualism meeting these constraints. Section 7 imposes a fifth constraint: ensuring that duplicating functional organization preserves phenomenology. Section 8 takes up subset dualism, a theory that impressively satisfies four of the five constraints, flouting only the non-overdetermination constraint. Section 9 formulates delegatory dualism and explains how delegatory dualism succeeds where its predecessors fail in satisfying all five constraints. Section 10 answers objections. Section 11 takes stock.

## The causal exclusion problem

I will distill the first few constraints from the causal exclusion problem. We can formulate the problem using the following set of jointly inconsistent claims:

**Non-Physicality**: Every experience is non-physical.^6^

**Efficacy:** Experiences generally occur at times and cause physical events.

**Closure:** Every physical event *e* that is caused at a time *t* has at *t* a sufficient physical cause in *e*’s ancestry of immediate sufficient physical causes.^7^

**Non-Overdetermination**: It is not generally the case that experiences’ effects have both non-physical causes and concurrent causes from their ancestries of immediate sufficient physical causes.^8^

Since this problem is well-known, I will keep the motivation of its claims brief. Dualism is committed to Non-Physicality. Efficacy reflects the pre-theoretical commitment to experience causing behavior. Something like Non-Overdetermination is widely regarded as plausible, though the sources of its plausibility are disputed and often left implicit. I investigate the sources of its plausibility elsewhere.^9^ Closure’s motivation rests on alleged empirical support from the fact that, as they have progressed, physics and neuroscience have regularly found physical causes to explain effects within their domains and have never found a need to posit non-physical causes to explain such effects.^10^

For dualists in the market for constraints on theorizing, the causal exclusion problem yields an embarrassment of riches. Since Efficacy, Non-Overdetermination, and Closure are jointly inconsistent with dualism, at most two of them can be adopted as constraints on a dualist theory. Dualists often adopt a defensive strategy for solving the causal exclusion problem: they accommodate two of the claims and defend as not prohibitively costly the combination of dualism with the negation of the remaining claim.^11^ We could follow such dualists and adopt two of the three available constraints, leaving the third by the wayside. However, there is another strategy which will prove more fruitful for the purposes of constructing a dualist theory: rather than settle for two constraints, we can extract three from the exclusion problem if we weaken one claim just enough to avoid inconsistency. The next section implements this strategy by forgoing Closure in favor of a slightly weaker constraint.

## Observational closure

I confess that I am unimpressed by Closure’s empirical credentials.^12^ Admittedly, we have strong evidence for Closure over certain forms of dualism that would contravene it. For instance, we can safely rule out the view that non-physical experiences make physical systems that have them impervious to gravity. But not all Closure-violating forms of dualism conflict with our evidence. For instance, there is *quantum interactionist dualism* on which experiences are non-physical states that cause wave function collapses.^13^ And there is *ampliative interactionist dualism* on which (i) experiences are non-physical, (ii) they violate Closure by inducing in neural firing patterns small, albeit in principle detectable, deviations from physical laws, and (iii) the induced deviant effects are then amplified into behavioral effects through non-deviant causal chains that accord with physical laws. Such forms of dualism lie in the realm of theories whose distinctive observational predictions we are not yet in a position to check. To wield Closure against such theories would be to go beyond what its empirical credentials license.

Still, I think that it is reasonable to adopt something like Closure as a working hypothesis. And I think it is a worthwhile project to consider what form dualism should take if Closure’s empirical credentials turn out to be impeccable in future physics and neuroscience. If the project yields a promising form of dualism, it will thereby defang arguments from Closure against dualism. Likewise, if the project yields no such theory, those arguments will be bolstered. So, I will adopt the following as a constraint:

**Observational Closure**: There are no observable violations of Closure.

An observable violation of Closure is a violation of Closure that would be recognized as such by an experiment of the sort performed in physics or neuroscience that lies within the realm of nomic possibility, i.e. one whose execution is not forbidden by the laws of nature that hold in our world. Since Observational Closure allows for unobservable violations of Closure, it is weaker than Closure.^14^

Retreating from Closure to Observational Closure is admittedly not the only way to extract an additional constraint on dualism from the causal exclusion problem. For instance, one could adopt an even weaker constraint of respecting our current evidence for Closure. Indeed, I think that adopting that constraint and investigating views such as ampliative and quantum interactionist dualism that respect it while violating Observational Closure is another worthwhile project.^15^ It’s simply not the project of this paper.^16^

Let me forestall two worries about constraining dualism with Observational Closure rather than Closure. First, it might be thought that Observational Closure would in effect collapse into Closure under the weight of induction. The worry is that, because Observational Closure would provide strong inductive support for Closure, there is no way—short of abandoning the canons of induction—to respect Observational Closure without also respecting Closure.

My response is that we should wait and see. Sections 4 and 9 examine forms of dualism that respect Observational Closure while violating Closure. It will be evident upon examining them that the above worry, however much prima facie plausibility it has in the abstract, has little force against these theories. Perhaps all such theories will ultimately be ruled out on other grounds, clearing the way for the inference from Observational Closure to Closure. Even if so, no harm will come from proceeding cautiously by initially constraining our theorizing with Observational Closure rather than Closure.

An analogy serves to illustrate the operative methodology. The prevailing view of classical mechanics is that it should be formulated in a Galilean space–time that does not support absolute velocities rather than in a Newtonian one that does.^17^ Both formulations respect observational Galilean relativity, which entails that no global differences in absolute velocity are observable. Observational Galilean relativity—in concert with other theoretical desiderata, e.g., being comparatively parsimonious—is taken to support the Galilean formulation. However, it would be hasty to adopt the Galilean formulation on the basis of observational Galilean relativity in advance of considering other theories (e.g., Newton’s) that respect observational Galilean relativity. Inferring Closure from Observational Closure in advance of considering theories that respect the latter but not the former would be similarly hasty.

The second worry is that any theory which respects Observational Closure while violating Closure would have to be conspiratorial. This sort of worry also arises for some physical theories. In the following passage, Maudlin nicely articulates the worry as it arises for one such theory (Bohmian mechanics)^18^:

The ... ground of suspicion … raises its head whenever terms like ‘‘conspiratorial’’ or ‘‘concocted’’ … are used to describe a theory. The underlying notion is something like this: if one employs a certain structure in formulating a physical theory, then *ceteris paribus* one should expect that the structure will be accessible through physical interaction. Otherwise, the existence of the structure is being somehow ‘‘covered up’’ by some complex mechanism or adjustment of free parameters: the rest of the theory must be fixed, or delicately balanced, to ‘‘hide’’ the structure from view. In a natural, unconcocted theory one can expect the essential physical structures to *show themselves* to the determined observer. (2008: 164–5; emphasis his)

Applied to the present context, the worry is that, all else equal, we would expect any theoretical posits that violate Closure to violate Observational Closure—unless the posit is somehow objectionably concocted to avoid exactly that result.

The worry can be pressed by considering an unconscious physical system (U) that is isolated from non-physical influences (and hence devoid of Closure violations) along with a conscious physical system (C) that features Closure violations but none that is observable. Let L be the physical law^19^ that in our world determines (insofar as they are determined) the evolution and causal structure of physical systems that are unconscious and isolated from conscious causes. We may assume that U will both apparently and in fact conform to L. In that case, C will also apparently conform to L, lest it yield observable violations of Closure. However, these two appearances will admit of quite different explanations. C’s apparent obedience will be explained in part by the fact that it engages psychophysical laws which generate non-physical experiences that make non-redundant causal contributions to C’s evolution. In contrast, since U is unconscious, such laws have no role in explaining why it apparently obeys L—instead, there will be a different set of laws that help explain why U apparently obeys L. This leaves us with two sets of laws that yield systems which apparently obey L. In the absence of some sort of delicate selection of laws, we would not expect two different sets of laws to yield systems that apparently obey L.^20^ In this manner, positing violations of Closure that are unaccompanied by violations of Observational Closure seems to lead to an objectionable form of fine-tuning.

Immediately after voicing the worry in the passage quoted above, Maudlin writes:

This principle [that one can expect essential physical structures to show themselves to determined observers] has some prima facie plausibility, but its general plausibility clearly cannot outweigh the consideration of particular cases. And in the case… we have been discussing… *no particular effort or adjustment of parameters is made with the purpose of hiding* [the relevant structure.] Rather, one writes down the simplest dynamical equations… and it *turns out* [that the relevant structure] will not be empirically accessible. (2008: 165; his emphasis)

Similarly, I grant the prima facie plausibility of the worry but insist that it should not be used to rule out in advance particular theories that respect Observational Closure and violate Closure. On delegatory dualism, the empirical inaccessibility of Closure violations will not, as it were, be written in by hand. Instead, it will be a consequence of delegatory dualism’s meeting other well-motivated constraints in a natural way that it yields only unobservable violations of Closure. I will revisit this issue in Section 10.

## Two dualist theories that satisfy three constraints

Having adopted Efficacy, Non-Overdetermination, and Observational Closure as constraints, I now turn to construct two forms of dualism that satisfy them. It is not obvious how to go about constructing such theories. A hint can be gleaned from Koons & Bealer, who write:

Anti-materialism is alleged to be unable to accommodate… mental causation without violating the causal closure of the physical. But this is not at all clear when causal closure is formulated in its most plausible form…: for every physical event *e* that has a cause, there is a physical event *c* such that it is nomologically (or causally) necessary that if *c* occurs, *e* occurs. Suppose that physics... provides justification for... this weak… principle. But obviously this weak principle does not imply the following stronger… principle: for every physical event *e* that has a cause, there is a physical event c such that c is a sufficient *cause* of *e*. Failure to appreciate the distinction... has led... to the conclusion that mental causation is untenable in an anti-materialist setting. (2010: xix; emphasis theirs)

Notice that the stronger closure thesis demands causal sufficiency where the weaker closure thesis permits nomic sufficiency. What does this distinction come to? We can say that for x to *nomically suffice* for y is for x and the laws of nature to together entail y. And for x to *causally suffice* for y is for x to nomically suffice for y in virtue of its causal contribution to y.^21^

Now, there is some plausibility to the hypothesis that physics is a guide to nomic sufficiency, albeit one that is ill-equipped to distinguish between differences in causal structure that outrun differences in nomic structure. Witness the fact that the debate about whether there is any causation at the fundamental physical level takes place against a background of agreement about what physics says about the laws at that level.^22^

This suggests the following two-step strategy for constructing dualist theories that respect Efficacy, Non-Overdetermination, and Observational Closure. First, construe putative sufficient physical causes of experiences’ effects as mere nomic guarantors of those effects, i.e. construe those physical states that Closure-respecting views deem to be sufficient causes of experiences’ effects as states that nomically suffice for those effects without qualifying as sufficient causes of them. Second, assign experiences a non-redundant role in bringing about those effects.

Recall L, the physical law that determines the evolution and causal structure of isolated unconscious physical systems. As Section 3 illustrated, respecting Observational Closure evidently requires conscious physical systems to apparently obey L. As it stands, the proposed strategy does not rule out conscious physical systems that manifestly violate L. To close this loophole, we can understand ‘nomically guarantee’ as short for nomically guarantee in a manner that apparently obeys L.

We can flesh out this strategy in two ways, corresponding to two forms of non-redundant causation: joint causation and preemption.^23^ These forms of causation are commonplace. When one sets an anvil and a brick on a scale, they jointly cause a certain measurement. When a major and sergeant order their troops to advance, the former’s command preempts the latter’s.^24^ These notions can also be clarified through their characteristic subjunctive signatures: when states jointly cause an effect, if one of those states had been absent, the others would not have produced the effect (cf. Won (2021)). And when one state preempts another, the preempted state does not cause the effect but it would have caused the effect had the preempting state not done so.^25^

Two existing proposals can be read as fleshing out this strategy with joint causation. One is advocated by Won (2021). He proposes to combine a dualist (‘emergentist’) view of mental properties with a joint causation model. On his proposal, mental states nomically supervene on physical states, which nomically suffice for certain physical effects. But the physical base would not produce the nomically guaranteed effect in the absence of the accompanying mental state—rather, it is only by causing the effect together that either the mental state or its physical base cause the effect at all. A similar model is also proposed by Lowe (2000, 2003). His model involves complications such as simultaneous causation and differences between event and fact causation that will not concern us here. Neither proposal specifies candidates for psychophysical laws.

To develop the strategy, we can formulate a view adapted to the present context that is in the spirit of Won’s and Lowe’s proposals. In particular, let’s consider *cooperative (interactionist) dualism*, which adds to dualism the claim that each experience *x* is accompanied by some physical state *y* such that:

- *y* is a nomic guarantor and putative sufficient physical cause of some physical event *e*,
- *x* is not a sufficient cause of *e*,
- *y* is not in *e*’s ancestry of immediate sufficient physical causes, and
- *x* and *y* jointly cause *e*.

Likewise, to develop the strategy with preemption, let’s consider *preemptive (interactionist) dualism*, which goes beyond dualism by claiming that each experience *x* is accompanied by some physical state *y* such that:

(i) *y* is a nomic guarantor and putative sufficient physical cause of some physical event *e*, and
(ii) *x* both causally suffices for *e* and preempts *y* from doing so except by way of *x*.^26^

The causal structures posited by these views can be visualized as follows^27^:
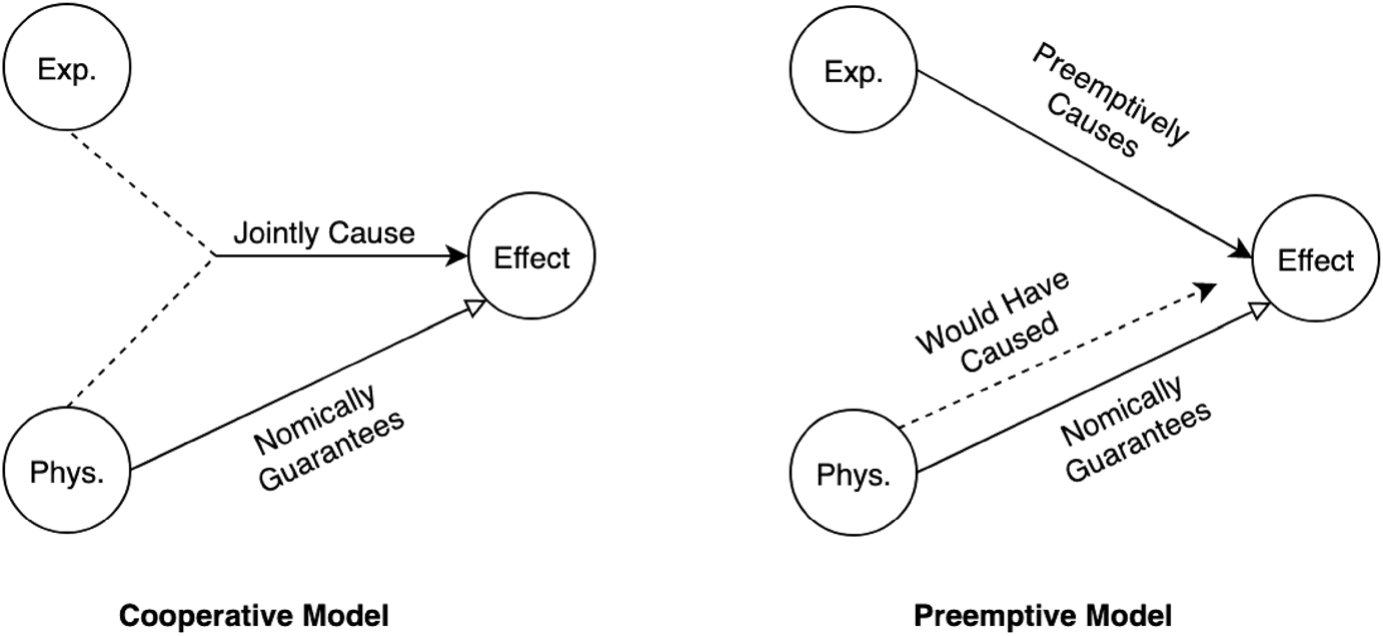


To illustrate how these views satisfy Efficacy, Non-Overdetermination, and Observational Closure, let’s consider (without loss of generality) a simple case of pain causing wincing. On both cooperative and preemptive dualism, the pain is a non-physical state that causes the wincing and which is accompanied by a physical state that nomically guarantees the wincing. So, the pain satisfies Efficacy. Since the wincing is nomically guaranteed in a way that apparently conforms to the physical laws regulating unconscious systems, there are no observable violations of Closure here. So, Observational Closure is satisfied. Moreover, cooperative dualism deems the pain a joint cause of the wincing, while preemptive interactionism deems the pain a preemptive cause of the wincing. If any physical state accompanying the pain lies in the wincing’s ancestry of immediate sufficient physical causes, the physical nomic guarantor does. But that state does not on either of cooperative or preemptive dualism. On either, the pain causes the wincing in the absence of such a sufficient physical cause; so Non-Overdetermination is satisfied.

Having identified two forms of dualism that satisfy Efficacy, Non-Overdetermination, and Observational Closure, I propose to add some further constraints to the mix.

## The upward systematicity constraint and challenge

The next constraint is adopted from Chalmers (1996). While discussing how to go about seeking a non-reductive theory of consciousness, Chalmers writes:

… the cornerstone of a theory of consciousness will be a set of psychophysical laws governing the relationship between consciousness and physical processes. These laws will tell us just what sort of experience will be associated with different sorts of physical process… An ultimate theory will not leave the connection at the level of “brain state X produces conscious state Y” for a vast collection of complex physical states and associated experiences. Instead, it will systematize this connection via an underlying explanatory framework, specifying simple underlying laws in virtue of which the connection holds… Ultimately, we will wish for a set of fundamental laws. Just as physicists seek a set of basic laws simple enough that one might write them on the front of a T-shirt, we should expect the same for a theory of consciousness. (1996: 213–4)

This passage nicely captures the *Upward Systematicity*^28^ constraint of specifying a simple set of fundamental psychophysical laws that yields the experiences in our world as a function of physical states in our world.^29^ Since cooperative and preemptive dualism do not propose any such laws, they do not meet this constraint. Thus, upon adopting Upward Systematicity, we must conclude that these views are either false or incomplete. This leaves open the possibility that one of them can be developed in a way that satisfies Upward Systematicity. Indeed, I will show that this is the case, as delegatory dualism will be a development of preemptive dualism.

The main reason for adopting Upward Systematicity is that we can be reasonably confident that it will be met by the true and complete dualist theory of experience, if there is such a theory. But there is another reason for trying to construct dualist theories that meet it: it can be leveraged into a challenge to dualism. The *Upward Systematicity challenge* is to provide at least a toy model of a dualist theory that satisfies Upward Systematicity. This challenge is urgent because, left unanswered, it gives us a reason to doubt that any realistic dualist theory satisfies Upward Systematicity. That, taken in concert with our confidence that a true and complete dualist theory would satisfy Upward Systematicity, would then tell against dualism.^30^

The Upward Systematicity challenge has been neglected by both dualists and anti-dualists.^31^ Yet, in my view, it is the most daunting one facing dualists. At present, there is a dearth of proposed answers to it. Unlike the causal exclusion problem, it does not rely on a shaky empirical foundation. Unlike the interaction problem of explaining the possibility of phenomenal-physical causal relations, dualists cannot answer it by appealing to virtually any of the available theories of causation. Unlike the objection from parsimony, it cannot be met by contending that dualism’s posits are needed to explain first-personal data. Delegatory dualism will at once meet this challenge and satisfy Upward Systematicity.

## Downward systematicity: a challenge met and a constraint unimposed

To satisfy Upward Systematicity, a theory needs to specify physical-to-phenomenal laws. Experiences’ causal contributions to the physical domain lie outside the jurisdiction of such laws. Yet any such contributions would presumably also be backed by systematic fundamental laws. This suggests an additional constraint: the *Downward Systematicity constraint* of specifying fundamental laws of phenomenal causation that fix experiences’ causal contributions as a function of their (intrinsic or extrinsic) features.

Like Upward Systematicity, Downward Systematicity would be met by a true and complete dualist theory. However, options for meeting Downward Systematicity are, as far as I can tell, relatively independent of delegatory dualism’s other commitments. (Toy versions of such options are given below.) And I am not aware of any compelling reasons for taking one of these options over any of the others. So, to allow delegatory dualism to remain neutral between these options, I will not adopt Downward Systematicity as a constraint. But I will address a challenge it raises for dualist theories, including delegatory dualism.

It might seem that cooperative and preemptive dualism satisfy Downward Systematicity. After all, cooperative dualism holds that experiences contribute to their effects by jointly causing them with the help of accompanying physical states. And preemptive dualism holds that experiences contribute to their effects by causing them in a manner that preempts would-be physical causes. However, while these tenets tell us something about what form phenomenal causation takes, each is compatible with a wide range of candidate fundamental phenomenal-to-physical laws. Thus, without supplementation, cooperative and preemptive dualism do not meet Downward Systematicity, as they do not come close to specifying a function from experiences’ features to their causal contributions.^32^

Just as Upward Systematicity lends to the Upward Systematicity challenge, so too does the Downward Systematicity lend to the *Downward Systematicity challenge*: provide at least a toy model of a dualist theory that satisfies Downward Systematicity.^33^ Left unmet, this challenge raises doubts about whether dualist theories can satisfy Downward Systematicity.

The Downward Systematicity challenge is less urgent as a threat to dualism than the Upward Systematicity challenge. For epiphenomenalist dualism is an already defended form of dualism that satisfies Downward Systematicity and *a fortiori* meets the Downward Systematicity challenge. On epiphenomenalist dualism, it’s a fundamental law^34^ that no experience causes anything.^35^ While this may be a degenerate law, it answers the Downward Systematicity challenge. However, this answer to the challenge is unavailable to dualist theories that satisfy Efficacy. Since we are seeking such a theory, we are under pressure to meet the challenge in some other way.

The following is a natural strategy for meeting this challenge: specify a toy law of phenomenal causation that qualifies a property (quantity, or relation) as a “causal magnet” that biases experiences towards causing states with that property. It is open to dualists to construe these laws as either contingent causal laws or as principles that systematically encode essential causal powers of experiential properties.^36^ In formulating laws of phenomenal causation, it will be useful to appeal to *candidate effects*, where a state *x* is a candidate effect of an experience relative to a given law if the other laws do not forbid that experience from causing *x* when applied to that experience and its circumstances. With this notion in hand, the strategy could be implemented by appealing to a law of the following form:

**Toy Law Schema**: Every experiential property *E* is such that whenever a subject instantiates *E*, their instantiating *E* causes whichever candidate physical effect maximizes *ψ* for that subject, where ‘*ψ’* is a schematic variable ranging over quantities.

Applications of this schema—ones that result from plugging in a quantity for *ψ*—yield toy laws of phenomenal causation.

Toy laws of phenomenal causation will qualify as especially promising answers to the Downward Systematicity challenge to the extent that they, or foreseeable refinements of them, harbor the potential to systematically generate rich causal profiles for experiences in creatures like us. Some such laws result from plugging in the following for *ψ* in the Toy Law Schema:

- Φ (the quantity of “integrated information”^37^),
- (thermodynamic or informational^38^) entropy,
- naturalness,^39^
- rationality,^40^
- match between phenomenal and neural structure,^41^ and
- match in representational content between experiences and neural representations.^42^

Still other toy laws could be generated by schemas that are couched in terms of the conservation or minimization of such quantities rather than their maximization.^43^

The resulting instances of Toy Law Schema are simple—any could be written on the front of a T-shirt. Perhaps you have worries that render some of these non-starters, even as toy laws. But it is quite plausible that at least some of them would generate rich causal profiles for experiences in subjects who are like us in physical respects. That is enough to lay the Downward Systematicity challenge to rest. The tasks of refining and adjudicating between candidate phenomenal causation laws such as these is the stuff of an unlaunched dualist research program that I will not pursue here.

## Organizational invariance

The fifth and final constraint is Chalmers’s (1996: Ch. 7; 2010: 23–25) (*principle of*) *Organizational Invariance*, according to which duplicating (fine-grained)^44^ functional organization duplicates phenomenology.^45^ Organizational Invariance is restricted to nomically possible worlds. So, Organizational Invariance entails the nomological impossibility of functional zombies (unconscious functional duplicates of actual conscious individuals) and functional inverts (individuals with experiences that are phenomenally inverted relative to experiences of actual individuals with the same functional organization); but it is consistent with their metaphysical possibility.

By way of motivation for Organizational Invariance, here is a brief rendition of Chalmers’s argument for it. We start by supposing for *reductio* that Organizational Invariance is false. In that case, there will be nomically possible functional duplicates F_1_ and F_2_ that radically differ in phenomenal respects. Perhaps F_1_ is a conscious creature while F_2_ is its unconscious or phenomenally inverted silicon counterpart. We can stipulate that F_1_ is well-functioning, in that she is reliably introspecting and reporting on her experiences under excellent conditions for such activity. Next, imagine a scenario in which F_1_ gradually transforms into F_2_ with functional organization preserved throughout.^46^ The subject of this transformation will undergo significant phenomenal changes. Yet given the preservation of functional organization, she will not notice or report these significant changes. Similarly, if we make her have “dancing experiences” by oscillating between states in different portions of the gradual process, she will not notice or report these changes in her experience.

Thus, from the supposed falsity of Organizational Invariance, we have derived the nomic possibility of a being that fails to notice significant changes in her experience, despite sharing the functional organization of a well-functioning conscious creature. This consequence tells against the initial supposition that Organizational Invariance is false. For, as Chalmers observes:

It is a central fact about experience, very familiar from our own case, that whenever experiences change significantly and we are paying attention, we can notice the change; if this were not the case, we would be led to the skeptical possibility that our experiences are dancing before our eyes all the time. This hypothesis has the same status as the possibility that the world was created five minutes ago: perhaps it is logically coherent, but it is not plausible. (2010: 24)

Given the plausibility of this argument, Organizational Invariance is a plausible constraint on theories of consciousness.^47^ Of course, it can be resisted in various ways. Chalmers (1996) anticipates and—successfully, I think—answers a slew of objections from the practical impossibility of the requisite transformation, from the charge that the argument would overgeneralize, from its having a soritical form, and from mild inversions, *inter alia*. I will not rehash that dialectic here.

Having motivated Organizational Invariance, I turn to its status as a constraint. As Chalmers argues, it is best seen as a constraint on fundamental psychophysical laws rather than as a candidate for such a law. In particular, it can be seen as imposing a constraint on fundamental laws satisfying Upward and Downward Systematicity: either there is a fundamental phenomenal causation law that induces functional differences wherever there are phenomenal differences, a fundamental physical-to-phenomenal law that allows phenomenal differences only where there are functional differences, or there is a set of fundamental physical-to-phenomenal and phenomenal causation laws that jointly preclude Organizational Invariance’s violation. Neither cooperative nor preemptive dualism proposes a fundamental psychophysical law; nor do they say anything about the source of phenomenal differences or the consequences of functional duplication. Consequently, without development, neither theory satisfies Organizational Invariance. In what follows, I will focus on theories that use a physical-to-phenomenal law to satisfy Organizational Invariance.

## Subset dualism

To set the stage for introducing delegatory dualism and showing how it satisfies all five constraints, I will consider a related theory that satisfies four of them, namely the *subset dualist* theory proposed (but not endorsed) by Pautz (2010: 357–8; fn40). On my preferred formulation, subset dualism goes beyond dualism by appealing to:

**Subset Law**: If the causal profile of an experiential property *E* is a subset of the causal profile of a physical property *P*, then whatever has *P* also has *E*.^48^

According to subset dualism, Subset Law is fundamental and it determines the distribution of experiences.^49^ This implies that every instantiated experiential property has a causal profile that is a subset of some instantiated physical property’s causal profile. Setting aside views on which experiential properties bring with them novel causal powers that exceed the powers of accompanying physical properties, all parties will agree that instantiated physical properties somehow possess causal profiles that subsume causal profiles of instantiated experiential properties. How experiential and physical properties acquire those profiles is left up for grabs. The picture, as Pautz describes it, is one on which:

... up in Plato’s heaven conscious properties have certain... causal powers. Complex systems evolved that have physical properties whose causal powers appropriately match those of conscious properties… these physical properties bring conscious properties with them. (2010: 358)

Let’s consider how subset dualism respects four constraints. First, Subset Law systematically yields experiences as a function of physical states.^50^ Thus, by construing Subset Law as a fundamental principle, subset dualism satisfies Upward Systematicity. Second, given that experiential properties are instantiated and that their causal profiles match those of instantiated physical properties, experiences presumably cause physical effects. So, subset dualists can easily satisfy Efficacy. Third, given that Subset Law determines the distribution of experiences, experiences cause effects only if accompanying physical states cause those effects. Thus, provided that physical states respect Closure, subset dualism satisfies Closure and hence Observational Closure. Fourth, subset dualism can easily satisfy Organizational Invariance: on subset dualism, there is no way to vary phenomenology independently of functional organization, as subset dualism takes certain functional features to determine phenomenology.^51^

Impressively, then, subset dualism satisfies Efficacy, Observational Closure, Upward Systematicity, and Organizational Invariance.^52^ However, as Pautz notes, subset dualism requires widespread overdetermination. For example, whenever a pain causes wincing, subset dualism entails that there is a distinct physical state that also causes wincing.^53^ Indeed, subset dualism entails that experiences overdetermine *all* of their effects. Given that those effects generally have ancestries of immediate sufficient physical causes (some members of which are concurrent with the relevant experiences), subset dualism thereby leads to violations of Non-Overdetermination.

## Delegatory dualism

Where does this leave us? It leaves us with two dualist theories—cooperative and preemptive dualism—that satisfy Efficacy, Observational Closure, and Non-Overdetermination but not Organizational Invariance, and with one dualist theory—subset dualism—that satisfies Efficacy, Observational Closure, Upward Systematicity, and Organizational Invariance but not Non-Overdetermination. We have yet to encounter a theory that meets all five constraints. But we can arrive at such a theory by synthesizing elements of the theories that satisfy some of them. The strategy is to:

1. Identify features of cooperative and preemptive dualism that enabled them to satisfy Non-Overdetermination without violating Efficacy or Observational Closure,
2. Identify features of subset dualism that enable it to satisfy Upward Systematicity and Organizational Invariance, and
3. Build those features into a single theory.

Regarding (1), it is by positing non-redundant forms of phenomenal causation (coupled with nomic guarantors) that cooperative and preemptive dualism satisfy Non-Overdetermination without violating Efficacy or Observational Closure. Regarding (2), it is by exploiting phenomenal-physical matches in causal profiles to determine the distribution of experience that subset dualism satisfies Upward Systematicity and Organizational Invariance.

We can construct a theory with these features as follows. Let a *default* causal profile be one that a property has conditional on the absence of non-physical, phenomenal interference. For physical states, default causal profiles will be what results from applying physical laws and not applying laws of phenomenal causation. *Delegatory dualism*, then, is a theory with the noted features. It is a version of preemptive dualism that appeals to:

Subset Law*: If the causal profile of an experiential property *E* is a subset of the default causal profile of a physical property *P*, then whatever has *P* also has *E*.

**Delegatory Law**: Whenever a subject instantiates an experiential property *E* and a physical property *P* such that *E*’s causal profile is a subset of *P*’s default causal profile, *E*’s causal profile preempts the corresponding subset of *P*’s default causal profile.^54^

On delegatory dualism, the operation of Subset Law* determines the distribution of experience. This implies that every instantiated experiential property has a causal profile that is a subset of some instantiated physical property’s default causal profile. Like subset dualism, delegatory dualism leaves open how experiential and physical properties acquire their causal profiles. Different versions of delegatory dualism will explain how experiential properties acquire their profiles in different ways; some appeal to refinements of the toy laws of phenomenal causation described in Section 6.

We can now see how delegatory dualism satisfies the five constraints. Subset Law* is a simple principle that fixes experiences as a function of physical states. Thus, by construing Subset Law* as a fundamental psychophysical law, delegatory dualism satisfies Upward Systematicity.

Subset Law appealed to the causal profiles of physical properties. In contrast, Subset Law* appeals to the *default* causal profiles of such properties. This difference enables delegatory dualism to succeed where subset dualism failed in satisfying Non-Overdetermination. We have already seen how subset dualism violates Non-Overdetermination. Let us now see how delegatory dualism satisfies it.

To start, return to the major and sergeant case. The major has the power to make the troops advance by commanding them to do so. The sergeant has this power by default, i.e. in the absence of interference from the major. Thus, there is a match between the major’s causal powers and the default causal powers of the sergeant. Yet when both command the troops to advance, no overdetermination results: the major’s command preempts the sergeant’s, frustrating its default efficacy. Similarly, because it is couched in terms of default causal profiles, Subset* Law (unlike Subset Law) does not inevitably lead to overdetermination. Indeed, it is the function of Delegatory Law to ensure that preemption, rather than overdetermination, results when phenomenal causal profiles match default physical causal profiles.^55^

To illustrate, suppose (in accordance with Subset Law*) that pain is instantiated because its causal profile is a subset of (say) C-fiber firing’s default causal profile. And suppose that both profiles encode the production of pain behavior (wincing, avoidance of the noxious stimuli, shrieks, etc.). Then Delegatory Law dictates that C-fiber firing will “delegate” that causal responsibility to pain, i.e. it dictates that pain rather than C-fiber firing will cause pain behavior, thereby preempting C-fiber firing’s default assignment to cause pain behavior. Thus, pain behavior results from preemptive phenomenal causation rather than physical-experiential overdetermination.^56^ The resulting pain behavior will be a part of various more-complex physical events that C-fiber firing had a default assignment to help bring about as a joint cause. Pain will likewise undertake these causal responsibilities, thereby qualifying as a joint cause of those physical effects.^57^ Likewise, on delegatory dualism, sensory experiential properties with certain causal profiles (such as enabling subjects to identify and distinguish objects in their environments, influencing what subjects say in perceptual reports, disposing subjects to ask whether sensory experience can be explained in physical terms, etc.) will be assigned to physical states with corresponding default causal profiles. Delegatory Law will then dictate that the sensory experiences rather than the physical states to which they are assigned uphold their shared causal responsibilities, i.e. that sensory experiences rather than those physical states cause in a given circumstance whatever effects their (default) causal profiles encode for that circumstance. And so on for other types of experience.

By positing experiences as preemptive and joint causes of physical effects, delegatory dualism satisfies Efficacy. Moreover, provided that physical states do not by default cause observable violations of Closure, no such violations will ensue. Thus, delegatory dualism satisfies Observational Closure. Finally, like subset dualism, delegatory dualism can easily satisfy Organizational Invariance: on both views, functional features of physical states determine phenomenology; so, both views readily ensure that phenomenal variations will be accompanied by functional variations.

## Objections

I turn now to address some objections that readers have raised to delegatory dualism.

First, recall the worry that delegatory dualism’s contention that the physical domain is only observationally closed required a sort of fine-tuning. We are now in a position to see how delegatory dualism renders Closure violations empirically inaccessible by meeting other constraints in a natural way rather than writing in such empirical inaccessibility by hand. Like other forms of interactionist dualism, delegatory dualism must posit violations of Closure in order to respect Non-Overdetermination and Efficacy. The unobservability of these violations results from delegatory dualism’s Subset Law*. But Subset Law* is not written into the theory simply to render Closure violations unobservable. Instead, delegatory dualism accords Subset Law* the role of meeting two independently motivated constraints—Upward Systematicity and Organizational Invariance.

Second, it may be objected that delegatory dualism’s use of Delegatory Law is ad hoc. This worry requires development, as there are various notions of ad hocery. The charge could be that the postulation of Delegatory Law is unmotivated. My reply is that its postulation is motivated by the fact that (when taken with Subset Law* and dualism) it yields a simple dualist theory that satisfies the five constraints motivated over the course of the paper. The objection could be that Delegatory Law is introduced to solve a problem for a theory while failing to yield any testable predictions. My reply to that charge is that the theory does yield testable predictions: it would be falsified by an observed violation of Closure. The objection could be that Delegatory Law results in a theory that does not yield any testable predictions that differ from subset dualism’s testable predictions. That is true. However, this puts delegatory dualism in the same predictive boat as epiphenomenalist dualism, overdeterminist dualism, and physicalism. Yet the predictive equivalence of these theories is not a basis for sinking the boat or casting any of its passengers overboard. The charge could be that Delegatory Law reconciles a theory with data in a way we’d expect to be inadequate as further data come in. My response is that if there is an objection here, it should be unpacked in terms of whatever supposedly warrants that prediction. If positing Delegatory Law is objectionably ad hoc, the sense in which it is so remains to be specified.

Third, one might object that delegatory dualism allows experiences to affect what their physical bases cause. However, this requires experiences to affect the application of physical laws to their (the experiences’) bases. But this does not make sense. What would it mean for experiences—which are *property instances*—to exert cross-category causal influence on *laws*?

To address this objection, it will help to set experience aside for a moment and consider (candidate) physical laws that apply only in restricted circumstances. In various physical theories that posit conservation laws, those laws apply only to closed systems. In classical mechanics Newton’s first law applies to objects only when (or only insofar as) they are not subject to outside forces. In collapse interpretations of quantum mechanics (e.g. GRW), Schrödinger’s equation applies only when the wavefunction is not collapsing.^58^ For each of these laws, it is clear that concrete physical phenomena would in some cases block the application of the law to a system. But there is little temptation to say that the concrete physical phenomena would do so by causally influencing the law. (A better suggestion may be that laws by nature only have *ceteris absentibus* force and that they are sensitive to concrete phenomena that affect whether they apply in the same way that they are sensitive to concrete phenomena that affect what the laws dictate when they do apply.) Taking their cue from these examples, delegatory dualists can maintain that experiences (per Delegatory Law) qualify as interfering factors that block physical laws from conferring certain causal powers to experiences’ physical bases. At the same time, delegatory dualists can deny that experiences interfere with the application of physical laws by exerting causal influence on the laws themselves, just as one can deny that external physical forces affect the applicability of Newton’s first law via causal influence on that law. Although a deeper account of how concrete phenomena can block the application of laws might be desirable, providing such an account is not something that delegatory dualism is under a special obligation to provide.

Fourth, one might object that delegatory dualism renders pressing the question of why *any* experiential properties are instantiated. After all, there presumably could have been worlds in which the distribution of physical properties and their causal powers did not yield a suitable match with any experiential property’s causal powers. In such a world, Subset Law* would not have generated any experiences. In response, delegatory dualism can render this explanatory challenge less pressing by countenancing a large and varied ensemble of experiential properties, many of which are uninstantiated for want of a suitable causal match with physical properties. For the more experiential properties there are, the less striking it is that some of them meet the instantiation conditions imposed by Subset Law*. Further, as it stands, the challenge does not reveal a distinctive difficulty for delegatory dualism, as it can be generalized to challenge a wide range of views. Given that only a small portion of metaphysically possible physical states at least nomically suffice for experience, we can ask: why do some such states rather than no such states obtain in our world? This question arises without an obvious answer on rival dualist and physicalist views no less than on delegatory dualism. Similarly, given that the laws of nature apply to only a small portion of the metaphysically possible states, we can ask why do some of those states obtain in our universe—for instance, why aren’t we in a stillborn world with Newtonian laws and quantum mechanical initial conditions?^59^ In light of these general problems, the proposed challenge would require much more development to provide evidence that delegatory dualism suffers an explanatory disadvantage.

Finally, there is what I regard as the most pressing objection to delegatory dualism—at least on the assumption that positing physical-experiential overdetermination is problematic. The objection is that although delegatory dualism avoids overdetermination, it posits a preemptive causal structure that inherits the theoretical vices of physical-experiential overdetermination. Developing and answering this objection is the topic of a companion work in progress.^60^ Here, I will restrict myself to noting two vices of overdetermination that do not infect delegatory dualism’s preemptive posit. This will provide reason to think that delegatory dualism’s preemptive posit is at least less costly than that of physical-experiential overdetermination.^61^

One vice of systematic experiential-physical overdetermination is that it seems to involve a striking but unexplained coincidence.^62^ In particular, it seems to involve such a coincidence between which experiential and physical properties are co-instantiated and which such properties share causal powers. This vice also attaches to some forms of systematic experiential-physical preemption, namely those that seem to posit such a striking but unexplained coincidence. But this vice does not afflict delegatory dualism. While delegatory dualism countenances a striking coincidence between which properties are co-instantiated and which share causal powers, it also explains the coincidence: per Subset Law*, experiential properties are co-instantiated with certain physical properties precisely because their causal powers match.^63^

Another vice of experiential-physical overdetermination is that it involves additivity failure whereby effects we’d expect to be additive functions of their causes’ contributions turn out to sum to less than the sum of those contributions.^64^ This vice does not attach to preemption: in contrast to overdetermination, preemption does not characteristically involve multiple causal contributions to a single effect, much less ones that violate additivity. When two rocks are thrown and one preempts the other from shattering a window by getting there first, we do not expect the preempted rock to add to the force of impact. Similarly, if an experience preempts a physical state’s causal contribution, we would not expect to find an effect that is somehow a sum of the experience’s causal contribution and the preempted physical state’s would-be causal contribution rather than just the effect that the physical state would have produced on its own. Thus, delegatory dualism escapes this vice as well.

## Conclusion

While my main aim has been to motivate a set of constraints and show how delegatory dualism satisfies them, I will conclude by stating an argument for delegatory dualism that is suggested by these constraints:

**Dualism:** Experiences somehow arise from physical states, despite being neither identical with nor grounded in such states.

**Efficacy:** Experiences occur at times and generally cause later physical events.

Therefore, interactionist (rather than epiphenomenalist) dualism is true.

**Observational Closure**: There are no observable violations of Closure.

Therefore, (e.g. quantum and ampliative) interactionist dualist theories that lead to observable violations of Closure are false. Instead, what’s true is an interactionist dualist theory on which experiences either overdetermine their effects or non-redundantly cause only unobservable violations of Closure, as on cooperative and preemptive dualist theories.

**Availability**: The only remaining available theories that satisfy Upward Systematicity and Organizational Invariance are subset dualism and delegatory dualism.

Therefore, we should accept subset or delegatory dualism over rival views.

**Non-Overdetermination**: It is not generally the case that experiences’ effects have both non-physical causes and concurrent causes from their ancestries of immediate sufficient physical causes.

Therefore, since subset dualism leads to overdetermination of the sort banned by Non-Overdetermination while delegatory dualism does not, we should accept delegatory dualism.

I have not defended dualism here. But I have argued for it elsewhere.^65^ Having combed the literature for other dualist theories that satisfy Efficacy, Observational Closure, Upward Systematicity, and Organizational Invariance and found none, I accept Availability.^66^ The remaining premises have been motivated as constraints on dualist theorizing over the course of the paper.

## Footnotes

1 For a grounding formulation of dualism, see, e.g., Chalmers (2015: 248).

2 We can think of (forward-looking) causal profiles as mappings from circumstances of instantiation to effects or probability distributions over candidate effects.

3 Cf. Bennett (2008: fn4). I’ll use ‘overdetermination’ throughout as shorthand for discussing causal structures that feature an effect with both a sufficient cause and a distinct cause, neither of which makes its contribution to the effect via the other. While this use accords with some standard uses of ‘overdetermination’, it contrasts with ones that build into the notion of overdetermination that it involves more than one sufficient cause, that it is analogous to paradigm cases such as firing squad cases, and/or that it is problematic. On the adopted use, it is thus substantive, even if plausible, to claim that widespread overdetermination is a problematic posit. As is often noted in the literature, which use of ‘overdetermination’ is adopted is just a terminological matter—see, e.g., Bennett (2003: 474).

4 See, e.g., Chalmers (2010: 21; 2018) and Vaassen (2024a: 54); cf. Cutter & Crummett (forthcoming).

5 E.g., see Malcolm (1968), Bennett (2008), Kim (1989, 2005: Ch. 2), and Papineau (2002: Ch. 1).

6 I use ‘non-physical’ to mean neither physical nor grounded in anything physical. I remain neutral between the standard definitions of ‘physical’ and understand grounding broadly to encompass all explanatory relations that are neither causal nor nomic whereby the explanans metaphysically necessitates the explanandum. Those who prefer to construe grounding as a sentential operator are invited to read the discussion in terms of their preferred notion.

7 Cf. Kim (2011: 112) and Lowe (2000, 2003). To accommodate quantum indeterminacy, Closure’s appeal to sufficiency may be replaced with an appeal to objective probability-fixing. To accommodate densely ordered causal structures, Closure may be refined to claim: for every physical event *e* that is caused at a time *t*, there is a physical event *p* at *t* such that *p* causally suffices for *e* along a path containing only physical events. For simplicity, I forgo these refinements in what follows.

8 These formulations of Closure and Non-Overdetermination are, per their appeal to ancestry, more complicated than some standard formulations. As Lowe (2000, 2003) shows, standard formulations of these claims are inapt, as they are consistent with interactionist dualist views. Such views will be important below; hence the need for the more complicated formulations.

9 See Saad (2019a: 162–173).

10 For the most developed empirical case for the causal closure of the physical domain, see Papineau (2002: Appendix).

11 Defenders (if not outright proponents) of epiphenomenalist dualism—which denies Efficacy—include Chalmers (1996: Ch. 4–5; 2010: 130–2), Gertler (2019), Jackson (1982), Kim (2005), and Robinson (2006, 2019); cf. Gibb (2015). Defenders of overdeterminist dualism include Fumerton (2013: Ch. 7), List & Stoljar (2017), Meixner (2004), Mills (1996), Kroedel (2015: §5; 2020: 171, 175), Vaassen (2024b) and White (2018). (As noted, in contrast to some uses in the literature, my stipulated notion of overdetermination leaves open whether overdetermination is problematic. So, in my terminology, ‘overdeterminist dualism’ is not a pejorative label, though the label will understandably be disavowed by defenders of the labeled position who use ‘overdetermination’ pejoratively.) Defenders of Closure-violating dualism include Elitzur (1989), Eccles et al. (1977), Foster (1991: Ch. 6), Swinburne (2013: Ch. 4), and Stapp (1993); cf. Bealer (2007), Chalmers (2010: 128), Libet (1994), and Lowe (2003). Descartes arguably held that non-physical minds affect the direction of bodily motions (McLaughlin, 1993); if so, then he advocated such a form of dualism. For defenses of each of these three standard forms of dualism by a physicalist, see Lycan (2009).

12 Others who have challenged Closure’s empirical credentials or the use to which physicalists put it include Bishop (2006), Cartwright (2010), Chalmers (2010: Ch. 5, fn24, fn26), Dimitrijević (2020), Garcia (2014), Gibb (2015), Koons and Bealer (2010), Lowe (2000), Montero (2003), Ney (2016), Saad (2018), and Tiehen (2015). For a proposal concerning how to run the causal argument with a weaker and more easily defended closure premise than is typically assumed, see Saad (forthcoming*b*).

13 See Chalmers (2010: 127–9), Chalmers and McQueen (2021), Stapp (1993), and Wigner (1961).

14 For discussion of how mental causation might be invisible to scientific investigation, see Lowe (2000, 2003).

15 Ampliative interactionist dualism violates Observational Closure by definition. Quantum interactionist dualism violates Observational Closure because nomically possible tests distinguish different collapse interpretations of quantum mechanics, including quantum interactionist dualism (although these tests would be very hard to run in practice) (Albert, 1992: Ch. 5; Chalmers & McQueen, 2021), and because quantum interactionist dualism construes the collapse postulate that generates its distinctive observable predictions as a basic psychophysical law rather than as a physical law.

16 Quantum interactionist dualism does, however, incur distinctive costs and face formidable challenges. Challenges include the problem of reconciling the view with relativity, making sense of superpositions of (conscious) states, the quantum zeno problem, and the tails problem—see Chalmers & McQueen (2021; 2022) and references therein. Such costs include lacking appealing features of other interpretations of quantum mechanics (such as conservativeness, intelligibility, and locality), incompatibility with the exclusion argument against epiphenomenalist dualism (Saad, 2020a), and requiring supplementation beyond the collapse postulate to recover something like the causal role that common sense assigns consciousness (Chalmers, 2018: 38–9; Goff, 2019, Ch. 2). These challenges and costs give dualists reason to develop alternative forms of interactionism such as those that respect Observational Closure. (My thanks to a reviewer for pressing for details here.).

17 See Maudlin (2012: 65–6).

18 Since the content of Bohmian mechanics is not relevant here, I omit it. See Goldstein (2017) for an overview.

19 Or set of laws.

20 Compare Einstein’s (1905) famous objection to what was the orthodox understanding of Maxwellian electrodynamics that it “leads to asymmetries which do not appear to be inherent in the phenomena”.

21 For anyone who finds this distinction elusive, it may help to consider some cases that plausibly involve nomic sufficiency without causal sufficiency. First, consider a variation of Leslie’s (1991) symmetric universe in which two sides of the universe deterministically evolve as perfect mirrors of one another. Sally throws a rock. By symmetry, her doppelganger also throws a rock. Intuitively, Sally’s throw is nomically but not causally sufficient for her doppelganger’s. Second, consider an epiphenomenalist scenario where the laws allow pain to occur if and only if it is caused by a certain brain state, they require that brain state to cause pain and avoidance behavior, and they preclude pain from causing anything. In this scenario, pain nomically suffices for avoidance behavior but does not cause avoidance behavior. Third, some contemporary physical theories have bidirectionally deterministic laws. On these theories, some future states nomically suffice for some past states. If causes must precede their effects, then those future states are nomically but not causally sufficient for those past states. Fourth, suppose that some initial conditions of the universe are dictated by a law (cf. Albert (2004) and Chen (2020)). The law nomically suffices for those conditions. However, given that laws cannot themselves be causes, the law does not causally suffice for those conditions. (My thanks to a reviewer for requesting elaboration.).

22 E.g., see Ney (2012: §6), Papineau (2013), and Russell (1912).

23 I use ‘non-redundant causation’ as shorthand for causation without causal overdetermination. While this is a standard use (e.g., see Bernstein (2016)), some authors instead adopt a broader use that encompasses preemption and joint causation (e.g. see McDermott (1995)). Nothing substantive turns on this choice. However, there is a substantive question of whether preemption is relevantly like problematic overdetermination—see Sect. 10 for discussion.

24 The major-sergeant case is a variation of one given by Van Fraassen (Schaffer, 2000: fn15). Bealer (2007: 26–7) appeals to the latter in developing an account of mental causation.

25 One might doubt that there is a deep distinction between worlds in which experiences are joint causes vs. preemptive causes vs. overdetermining causes vs. epiphenomena. For instance, perhaps on a counterfactual theory of causation, some of these distinctions will turn out to be merely verbal ones based on an arbitrary decision about which system of weights for cross-world similarity to use in evaluating the relevant counterfactuals—see Pautz (forthcoming: fn30, §5). If this turned out to be so, it would detract interest from the varieties of dualism discussed in this paper along with much of the mental causation literature, though it would arguably be good news for dualism itself (Vaassen, 2024a). I think this deflationary outcome is avoidable, as I favor a view on which the most basic causal relations are deep joints in nature underwritten by laws of nature. But defending that view would take us too far afield. So, I will simply adopt what I take to be the working assumption of much of the mental causation literature that there are substantive facts of the matter about the causal structure of the physical-phenomenal nexus.

26 The proviso allows for the possibility that *y* causally suffices for *x* which in turn causally suffices for *e* while preempting *y* from causally sufficing for *e* along another path. Note, however, that preemptive dualism is not committed to this—e.g. *x* and *y* could instead be effects of a common cause, neither of which causes the other. Delegatory dualism will turn out to be a form of preemptive dualism that inherits this degree of freedom.

27 For discussion relevant to cooperative interactionism, see Gibb (2015), Lowe (2000: 576–7; 2003: 151–2), and Kim (1989: 86). Bealer (2007) develops a non-reductive account of mental causation that construes mental-to-mental and mental-to-physical causation as a form of preemption (*ibid*: fn2); cf. Bernstein (2016: 27) and Yablo (1992). Bealer’s account is underexplored but too intricate to explore here.

28 This constraint is—like the Downward Systematicity constraint introduced below—so called after the convention in the mental causation literature of placing representations of mental states above representations of physical states in diagrams. For recent discussion of constraints along the lines of Upward Systematicity, see Bennett (2021) and Schaffer (forthcoming).

29 There are conceivable circumstances under which it would be reasonable for dualists to relax this constraint, e.g. to allow for fundamental psychophysical laws that are stochastic.

30 Analogous problems arise for some physicalists. Non-reductive grounding physicalists face the problem of providing at least toy physical-to-phenomenal grounding principles, while reductive physicalists face the challenge of providing toy reductions of experiences—cf. Byrne & Tye (2006: 253–4), Sider (2011: 117), and Pautz (2017: 356).

31 By ‘neglected’ I do not mean entirely ignored—for relevant suggestions, see Chalmers (1996: Ch. 8), Robinson (2014), and Bourget (2019).

32 The same goes for the versions and variations of these views cited above.

33 Cf. Davidson (1970), Elisabeth of Bohemia (1643), Lycan (2009: 557), and Kim (2005: 153–4). There is also a closely related challenge for cooperative and preemptive dualism of explaining why the presence of experiences makes specific differences to what physical states cause. For example, on preemptive dualism, the challenge is that of explaining why experiences not only cause certain effects but also preempt physical states from doing so. On delegatory dualism, this challenge is met through the postulation of a law (what I call ‘Delegatory Law’ below) that requires experiences to make certain causal contributions instead of their physical bases. On overdeterminist dualism, this challenge does not arise.

34 Or, if one prefers, the consequence of the fundamental laws plus the absence of any such law to the contrary.

35 On another common formulation, epiphenomenalist dualism instead denies that experiences cause *physical* effects. So formulated, it is open to epiphenomenalist dualists to posit phenomenal-to-phenomenal causation. Theorists who take that option are subject to the Downward Systematicity challenge no less than dualists who posit phenomenal-to-physical causation.

36 For dualist views that countenance essential causal powers, see White (2018) and Langsam (2011: Ch. 3); cf. Mørch (2018a: §6).

37 Φ plays a central role in the integrated information theory of consciousness advocated by Tononi (2004). For an overview of the theory and an informal characterization of Φ, see Mørch (2017). For different ways of formally specifying Φ, see Tegmark (2016).

38 See Shannon (1948).

39 See Lewis (1983) and Dorr & Hawthorne (2013).

40 See Saad (2019b; 2020b); cf. Chalmers (2020).

41 See Pautz (2013: §18.2; 2011: 404–5) for an overview of relevant empirical results concerning phenomenal-neural structural correlations, and Clark (1993) and Chalmers (1996: Ch. 6, §6.2; 2010: 20–3) for discussion of structural matches between experience and associated states. There is room for a structure-matching law running in the opposite direction, i.e. from neural states to experiences—see Robinson (2014: §7–9) for a suggestion along these lines; cf. Dalbey & Saad (2022: §5).

42 Cf. Chalmers (2010: 65).

43 Cf. Robinson (2014: §5–6) and Hart (1988: Ch. 9).

44 Following Chalmers (1996: 248), I will take fine-grained functional organization to be a level of functional organization that is “always… fine enough to determine behavioral capacities”.

45 For related arguments and cases previously proposed by other authors, see Chalmers (1996: 253, Ch. 7, note 19).

46 See Chalmers (1996: 257–8) for defense of the nomic possibility of such a transformation.

47 For a more rigorous rendition and reasons to think that the argument should aim to support a qualified version of Organizational Invariance, see Saad & Bradley (2022). For simplicity, here I will just work with an unqualified version of Organizational Invariance.

48 For defense of a closely related version of physicalism, see Shoemaker (2000). For a way of formulating physicalism that invokes subsets of causal powers, see Wilson (1999).

49 Subset Law may need to be refined to specify a causal relationship between *P* and *E* (e.g. that *P* causes *E*), to omit reference to causation in favor of its fundamental basis, or to concern properties’ causal profiles *with respect to physical effects*—similar qualifications apply to Subset Law* and Delegatory Law below.

50 Causal profiles of physical states qualify as physical.

51 A small loophole: since Organizational Invariance is couched in terms of fine-grained—but not maximally fine-grained—functional facts, there is room in logical space for a version of subset dualism on which some experiential properties’ causal profiles are distinguished only below that level of grain. However, the argument for Organizational Invariance tells against this view and there is no motivation for it. To close this loophole, subset dualists need only reject this version of their view. I ignore this loophole in what follows.

52 It’s clear from Pautz’s (2010: 357–8; fn40) discussion that subset dualism satisfies Efficacy and Upward Systematicity. He does not discuss (Observational) Closure or Organizational Invariance, though he does classify a generalization of subset dualism as a form of functionalism (*ibid*: 358).

53 The subset physicalist move of saying that the token causings and token causal powers of the two states do not result in (costly) overdetermination because those states are constitutively related seems unavailable here: on the subset dualist theory, because the non-physical pain and the accompanying physical state are constitutively independent, their token causings and token causal powers presumably are too.

54 N.B. we need not take ‘preempt’ as a primitive causal notion in this law. We can instead unpack the law’s content as follows: Whenever a subject instantiates an experiential property *E* and a physical property *P* such that *E*’s causal profile is a subset of *P*’s default causal profile, *any effect that falls within the scope of both profiles is caused by E rather than by P*.

55 One limitation of this comparison is that the major-sergeant case is an ordinary one in which we can understand preemption in terms of prosaic facts about the officers’ behaviors and the troops’ psychologies. In contrast, to grasp delegatory dualism’s preemptive posit, we need to understand how a law could—without mediation via prosaic mechanisms—result in experiences preempting rather than overdetermining their effects. With only this comparison to go on, one might worry that whether experiences preemptively cause rather than overdetermine their effects is a merely verbal issue. To allay this worry, I offer an alternative comparison. Consider the following, more-fanciful case introduced by Schaffer (2000) and described by Lewis (2004: 81): We can stipulate… that … the laws of magic state that what will happen at midnight must match the first spell cast on the previous day. The first spell of the day, as it happens, is Merlin’s prince-to-frog spell in the morning. Morgana casts another prince-to-frog spell in the evening. At midnight the prince turns into a frog. Either spell would have done the job, had it been the only spell of the day; but Merlin’s spell was first, so it was his spell that caused the transmogrification… Merlin’s spell was a preempting cause, Morgana’s was the preempted backup. But we stipulate also that the causal process from spell to transmogrification has no intermediate steps. Like Lewis and Schaffer, I take this to be an intuitive example of preemption that is underwritten by laws rather than prosaic mechanisms (see Schaffer (*ibid*) for an extended defense of this example from various objections). I take this to show that there is no general difficulty with understanding how laws could directly underwrite preemption; nor do I discern a distinctive difficulty for Delegatory Law on this score. (My thanks to a reviewer for pressing this worry.).

56 A minor complication: although Delegatory Law enables pain to preempt causal contributions from the C-fiber firing, pain still threatens to overdetermine causal contributions from C-fiber firing’s constituents. To vanquish this threat, we can refine Delegatory Law to say that E’s causal profile preempts the corresponding subset of P’s default causal profile along with any corresponding causal powers of P’s constituents. This will, for example, result in pain preempting causal contributions from molecules and atoms that help compose C-fiber firing rather than in the overdetermination of their effects.

57 Delegatory dualism is partly inspired by emergentist views on which emergent phenomena replace or attain metaphysical priority over their bases—see Humphreys (1996), Mørch (2018b: §4), and Seager (2010). On delegatory dualism, one can think of experiences’ causal powers as replacing or attaining priority over those of accompanying physical states.

58 See, e.g., Bell (1987: 202).

59 See Cutter & Saad (2023).

60 Materials for the response I favor can be found in my dissertation (2019a: 162–173).

61 Delegatory dualists could instead respond by arguing that overdetermination is not problematic and hence that there are no vices of overdetermination for delegatory dualism’s preemptive posit to inherit. This strategy could be pursued by appealing to accounts proposed by overdeterminist dualists for why physical-experiential overdetermination is unproblematic—see, e.g., List & Stoljar (2017), Kroedel (2020), Vaassen (2024b), and White (2018). Although I think that strategy is worth exploring, I will not pursue it here. One dialectical advantage of the strategy pursued in the main text is that it enables delegatory dualists to maintain that avoiding overdetermination is a point in favor of their theory over overdeterminist dualism. No such advantage is available to delegatory dualists who instead respond to the objection by denying that overdetermination is problematic.

62 See, e.g., Block (1990: 159), Lycan (1995: Ch. 1, note 5), Melnyk (2003: 169), and Sharpe (2015: 1120).

63 Subset dualism is free of this vice for the same reason, per its appeal to Subset Law.

64 See Bernstein (2016: 30–2), Kim (1992: 208), Paul and Hall (2013: 148–149, 157–158), and Saad (2017: §4).

65 See Saad (2018, 2021: §3.3; forthcoming*a*).

66 What about a cooperative (rather than preemptive) variant of delegatory dualism? Such a view would in effect co-opt delegatory dualism’s ability to satisfy the five constraints while replacing Delegatory Law with a principle of roughly the same form that yields widespread joint causation instead of preemption. The replacement principle would need to systematically divide causal labor between experiences and physical states in the production of their immediate joint effects. Such a principle would need not only to dictate how much each cause contributes to a given joint effect but also exactly what that cause contributes: a principle that splits the labor 50/50 between physical states and experiences will be inadequate if it fails to settle which half of the work each sort of the cause is to perform. It is difficult to specify simple principles of this sort that would divide labor even in one-off toy cases, e.g. of pain and C-fiber firing jointly causing wincing. This raises the worry that a replacement principle that divides labor in all cases where cooperative dualism requires division would need to be horrendously complicated. In any event, no such principle has been proposed; nor are there obvious candidates for such a principle. (My thanks to a reviewer for requesting elaboration.).
